# Decreased Exploratory Activity in a Mouse Model of 15q Duplication Syndrome; Implications for Disturbance of Serotonin Signaling

**DOI:** 10.1371/journal.pone.0015126

**Published:** 2010-12-15

**Authors:** Kota Tamada, Shozo Tomonaga, Fumiyuki Hatanaka, Nobuhiro Nakai, Keizo Takao, Tsuyoshi Miyakawa, Jin Nakatani, Toru Takumi

**Affiliations:** 1 Osaka Bioscience Institute, Suita, Japan; 2 Kyoto University Graduate School of Biostudies, Kyoto, Japan; 3 Graduate School of Biomedical Sciences, Hiroshima University, Hiroshima, Japan; 4 Kyoto University Graduate School of Medicine, Kyoto, Japan; 5 Frontier Technology Center, Graduate School of Medicine, Kyoto University, Kyoto, Japan; 6 Division of Systems Medicine, Institute for Comprehensive Medical Science, Fujita Health University, Aichi, Japan; 7 Section of Behavior Patterns, Center for Genetic Analysis of Behavior, National Institute for Physiological Sciences, Okazaki, Japan; 8 Japan Science and Technology Agent (JST), Core Research for Evolutional Science and Technology (CREST), Saitama, Japan; Tokyo Institute of Psychiatry, Japan

## Abstract

Autism spectrum disorders (ASDs) have garnered significant attention as an important grouping of developmental brain disorders. Recent genomic studies have revealed that inherited or *de novo* copy number variations (CNVs) are significantly involved in the pathophysiology of ASDs. In a previous report from our laboratory, we generated mice with CNVs as a model of ASDs, with a duplicated mouse chromosome 7C that is orthologous to human chromosome 15q11-13. Behavioral analyses revealed paternally duplicated (*patDp/+*) mice displayed abnormal behaviors resembling the symptoms of ASDs. In the present study, we extended these findings by performing various behavioral tests with C57BL/6J *patDp/+* mice, and comprehensively measuring brain monoamine levels with *ex vivo* high performance liquid chromatography. Compared with wild-type controls, *patDp/+* mice exhibited decreased locomotor and exploratory activities in the open field test, Y-maze test, and fear-conditioning test. Furthermore, their decreased activity levels overcame increased appetite induced by 24 hours of food deprivation in the novelty suppressed feeding test. Serotonin levels in several brain regions of adult *patDp/+* mice were lower than those of wild-type control, with no concurrent changes in brain levels of dopamine or norepinephrine. Moreover, analysis of monoamines in postnatal developmental stages demonstrated reduced brain levels of serotonin in young *patDp/+* mice. These findings suggest that a disrupted brain serotonergic system, especially during postnatal development, may generate the phenotypes of *patDp/+* mice.

## Introduction

Autism is a widely accepted neurodevelopmental disorder characterized by several major criteria, including impairments in social interaction, verbal and non-verbal communication difficulties, repetitive or rigid behavior, and restricted interest [1]. Autism spectrum disorders (ASDs) comprise autistic disorder, Rett's disorder, Asperger's disorder, and pervasive developmental disorder not otherwise specified (PDD-NOS) in DSM-IV. The prevalence of ASDs was recently estimated to have increased to 1 in 166 births [2]. According to previous studies of twins, these disorders are highly heritable because concordance rates for monozygotic twins (70–90%) are several fold higher than the corresponding rates for dizygotic twins (0–10%) [3]. Although there are many candidate genes for pathophysiology in ASDs, recent high-resolution genetic techniques revealed that a *de novo* copy number variation (CNV) is also a significant risk factor for ASDs, and is a more important risk factor than previously recognized [4].

In several candidate chromosomal regions with relevance for ASDs, duplication of human chromosome 15q11-13 is the most frequent chromosome rearrangement, found in 1–4% of ASDs patients [5]. Moreover, this region is an imprinting region where deletions or methylation abnormalities of paternal and maternal alleles leads to Prader-Willi syndrome and Angelman syndrome, respectively. In chromosomal duplication, these imprinting effects can also affect the resulting phenotypes. Maternal duplication of human chromosome 15q11-13 is believed to cause ASDs, whereas paternal duplication usually results in a normal behavioral phenotype [6]. However, recent findings suggest that maternal interstitial duplications may be less prevalent than previously assumed [7], and indeed paternal duplication of this region may lead to autistic-like behavior [7].

Various neurotransmitters may be involved in the pathophysiology of ASDs. Notably, serotonin (5-HT) concentrations in platelets of patients with ASDs are higher and more widely distributed than those of controls [8], [9]. However, this abnormality may not result in abnormal brain 5-HT levels because 5-HT generally does not penetrate the blood brain barrier (BBB). Nevertheless, a recent report showed that 5-HT could cross the BBB in rats [10]. Thus, the relationship between peripheral and central 5-HT in ASDs remains unclear, and might be more complex than established mechanisms would indicate. As further evidence for 5-HT abnormality in ASDs, administration of selective serotonin reuptake inhibitors (SSRIs) improved repetitive behavior which is a core symptom in ASDs [11].

In previous studies from our laboratory, using a chromosome-engineering technique, we generated mice with a 6.3 Mb duplication on mouse chromosome 7 that corresponds to human chromosome 15q11-13 [12], [13]. Furthermore, we found that *patDp/+* mice with a duplicated region derived from a paternal allele displayed several abnormal behavioral similarities to autistic phenotypes in two different strains, 129S6/SvEvTac (129S6) and C57BL/6J. However, no significant change has been observed on these behavioral tests for maternally duplicated (*matDp*/+) mice compared with wild-type (WT) mice [13]. In the present study, we conducted a comprehensive battery of behavioral tests in C57BL/6J *patDp/*+ mice to evaluate other behavioral abnormalities. Moreover, using *ex vivo* high performance liquid chromatography (HPLC), we performed quantitative analyses of biogenic amines in brains of adult and young mice to determine the role of brain monoamines in any measured behavioral abnormalities. The specific hypothesis tested was whether there is a relationship between specific behaviors and brain monoamines in *patDp*/+ mice, and the results suggest that disturbance of serotonergic signaling during development may cause abnormal behaviors in these mice.

## Results

### Decreased exploratory activity of *patDp*/+ mice

The results of a comprehensive set of behavioral tests are presented in Table S1. Compared with WT mice increased body weight and lower temperature in patDp/+ mice was observed. There is no difference between *patDp*/+ and WT mice in hot plate test, tail suspension test, and prepulse inhibition test (Table S1). Neuromuscular examination and rotarod experiments revealed that *patDp/+* mice possess normal motor coordination (Table S1).

**Figure 1:**
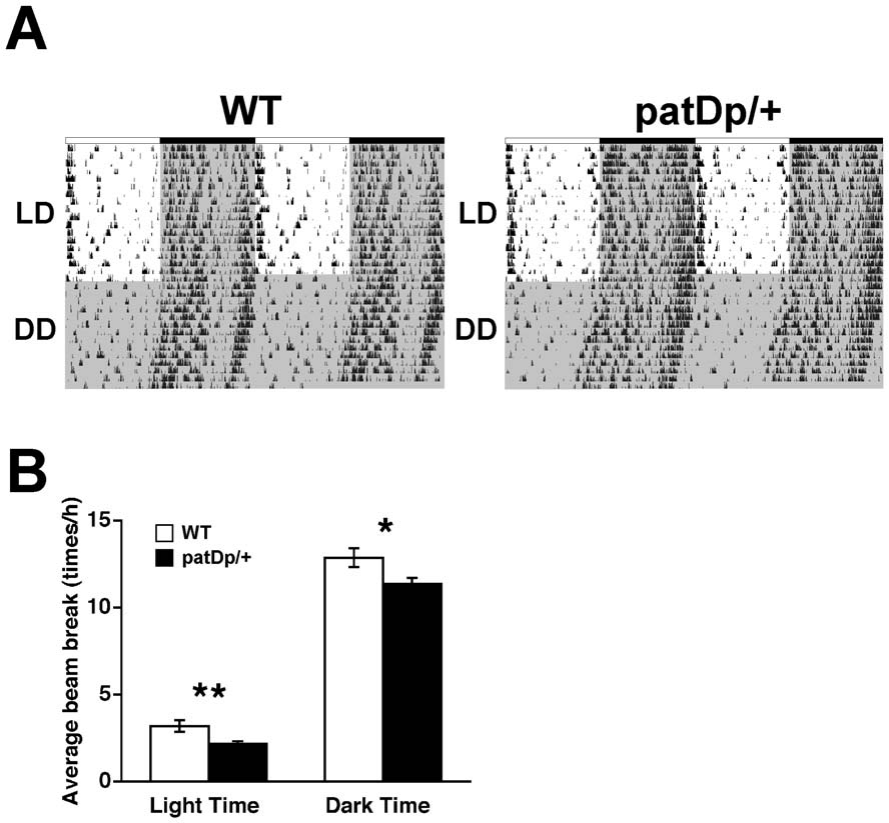
*patDp*/+ mice have decreased basal locomotor activity in their home cages. (**A**) Representative activity records from a WT mouse and a *patDp*/+ mouse. Light regimen: 17 d in LD followed by 15 d DD. (**B**) Activity difference during light phase (left) and dark phase (right) after 1 week in LD (*p*<0.01, 0.05, *t*-test, respectively). N = 4 for WT and 9 for *patDp/+*. Error bars indicate SEM. **, *p*<0.01, *, *p*<0.05.

To investigate the phenotypes of C57BL/6J *patDp/+* mice, we determined the circadian rhythms of locomotor activity in these mice compared with WT controls. Circadian rhythms in *patDp/+* mice warrant study because ASDs patients often exhibit disturbances of circadian rhythms and sleep [14]. To determine the free running periods of *patDp/+* mice, subjects were initially entrained for 2 weeks to a 12-hour light/dark cycle (LD) and then kept in constant darkness condition (DD) for subsequent 2 weeks. The circadian rhythm of locomotor activity in *patDp*/+ mice was not significantly different from WT mice (Fig. 1A). In fact, the circadian periods of locomotor activity displayed no significant difference between these genotypes (*t*_11_ = −0.63, *p*>0.05, *t*-test, data not shown). Recordings of basal locomotor activities in mice entrained for 1 week indicated that *patDp/+* mice exhibited decreased movement, even when habituated in their home cages (Fig. 1B; Light time; *t*_1090_ = −3.26, *p<*0.01, Dark time; *t*_1090_ = −2.41, *p<*0.05, *t*-test). These results suggest that *patDp/+* mice have spontaneously decreased locomotor activities and normal circadian rhythms.

**Figure 2:**
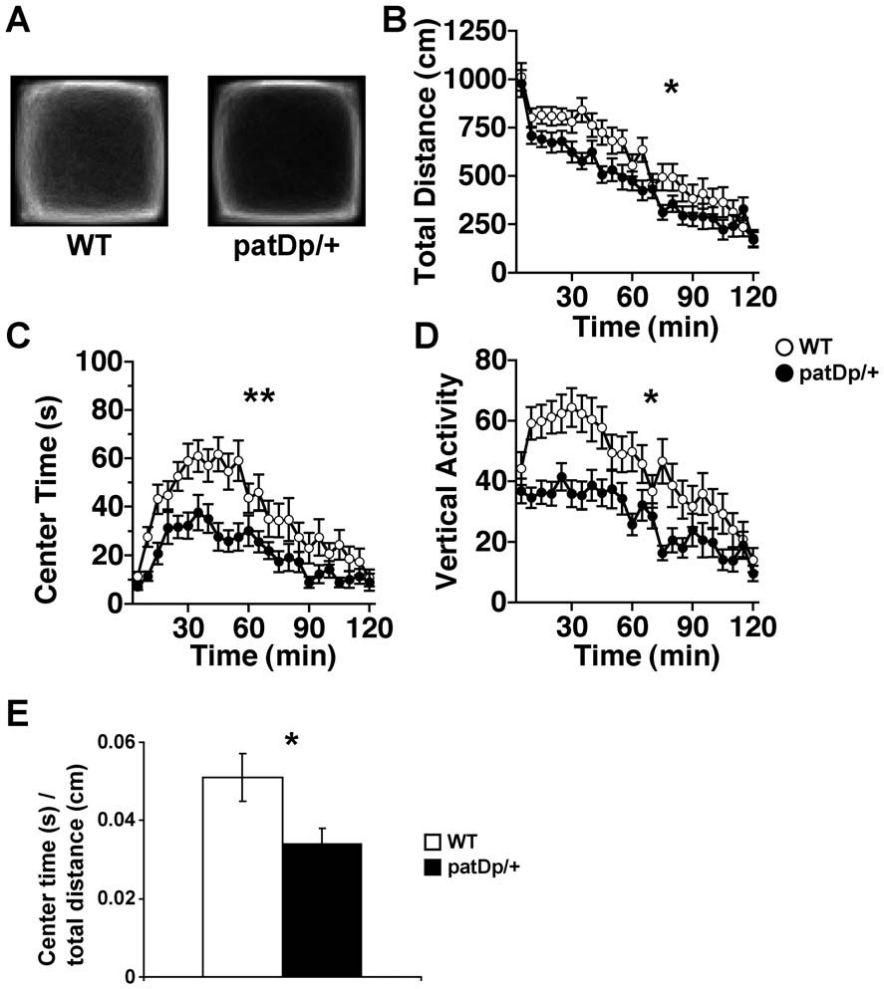
*patDp*/+ mice display decreased exploratory activity in the open field test. (**A**) Averaged trace images from each genotype. (**B**) Total distance traveled. (**C**) Time spent in the center area of the compartment. (**D**) Count of vertical activity. (**B**)–(**D**) Each data point represents the mean value in a 5-min segment. N = 22 for both genotypes. (**E**) The ratio of center time/total distance for the first 30 min was also calculated. Error bars indicate SEM. **, *p*<0.01, *, *p*<0.05.

During the 120 min test period of the open field test, *patDp*/+ mice exhibited less distance traveled (Fig. 2B; *F*_1,42_ = 5.84; *p = *0.02), less time spent in the center area accompanied by thigmotaxis (Fig. 2A; averaged trace images of each genotype) (Fig. 2C; time spent in center area; *F*_1,42_ = 11.61; *p<*0.01), and less vertical activity (Fig. 2D; *F*_1,42_ = 10.12; *p<*0.01) compared with WT mice. The ratio of center time/total distance for the first 30 min was also calculated as “anxiety index”. *patDp*/+ mice significantly showed the higher ratio than WT mice (Fig. 2E; *t*_42_ = 2.2, *p<*0.05, *t*-test). These results suggest that *patDp*/+ mice exhibited increased anxiety-like behavior and/or decreased locomotor exploration in novel environments.

**Figure 3:**
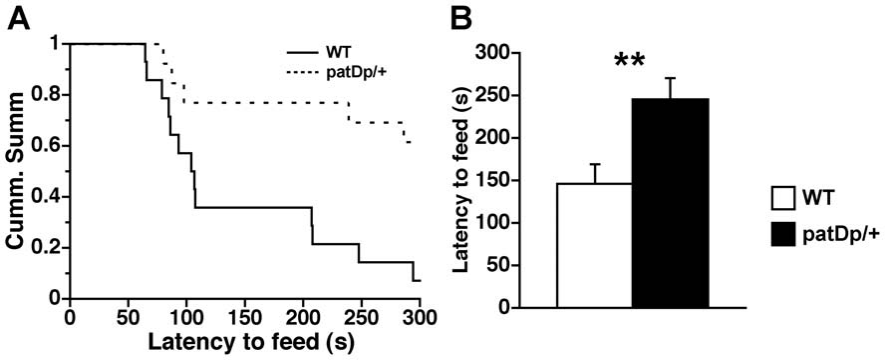
*patDp*/+ mice show longer latencies to feed in a novel environment. (**A**) Latency to feed in a novel environment (Cum. Summ  =  cumulative survival– i.e. the percentage of animals that have not eaten) (Kaplan-Meier survival analysis, Mantel-Cox log-rank test, *p*<0.01). (**B**) Average latency to feed in a novel environment. N = 14 for WT and 13 for *patDp*/+. Error bars indicate SEM. **, *p*<0.01.

We used the novelty suppressed feeding test [15] to determine whether *patDp/+* mice exhibit anxiety-like behaviors or are the lowered motivation caused by reduced activity. In this test, *patDp*/+ mice had longer latencies to feed than WT mice (Fig. 3B). Furthermore, most WT mice ate chow within 5 min, whereas approximately 60% of *patDp*/+ mice did not (Fig. 3A). Kaplan-Meier survival analysis and the Mantel-Cox log-rank test revealed that this was a significant difference (*p<*0.01). The amount of food consumed in the home cage after the test and body weight decreased (%) by 24 h food deprivation were not significantly different between *patDp*/+ and WT mice (*t*_25_ = 1.57, *p* = 0.13, *t*-test, for food consumed, *t*_25_ = 0.51, *p* = 0.61 for body weight, data not shown). These results indicate that *patDp*/+ mice have increased anxiety-like behavior and/or decreased activity, even when food deprivation opposes these effects.

**Figure 4:**
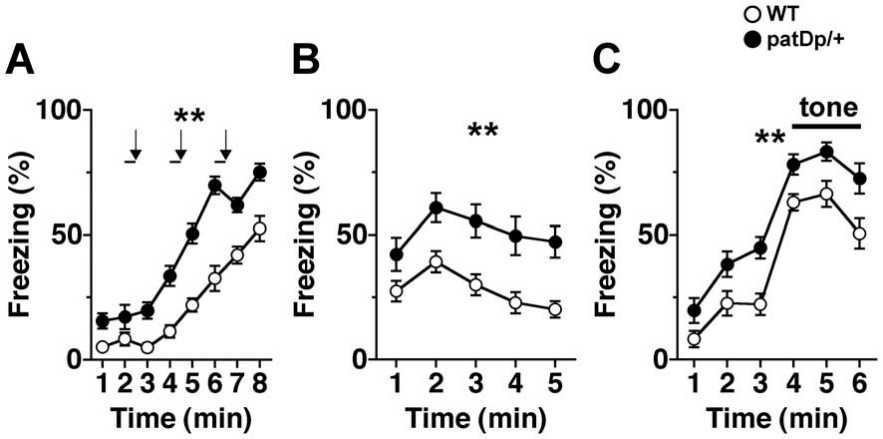
*patDp*/+ mice have higher basal freezing rates in the fear-conditioning test. (**A**) Immediate freezing during the fear-conditioning phase. Arrows and bars indicate unconditional stimuli (foot shock, 2 sec) and conditioned stimuli (white noise, 30 sec), respectively. (**B**) Contextual testing conducted 24 h after fear conditioning. (**C**) Cued test with altered context and pre-tone period are shown. N = 22 for both genotypes. Error bars indicate SEM. **, *p*<0.01.

Fear-like behavior was examined with the contextual and cued fear conditioning test. In this test, freezing responses to a foot shock in *patDp*/+ mice were similar to WT mice (Fig. 4A). However, basal freezing rates of *patDp*/+ mice in any condition tested, including both context and altered context with cues conditioning, were significantly increased compared with WT mice [Fig. 4A; *F*_1,42_ = 44.58; *p<*0.01, 4B; *F*_1,42_ = 16.25; *p<*0.01, 4C (pre-tone period); *F*_1,42_ = 8.49; *p<*0.01, (cued); *F*_1,42_ = 14.04; *p<*0.01]. These results may suggest that *patDp*/+ mice displayed fear-like behavior. Alternatively, since the basal freezing rates in *patDp*/+ mice are substantially higher before stress conditioning, these unusual phenotypes could represent decreased locomotor activity and/or increased novelty-induced anxiety.

**Figure 5:**
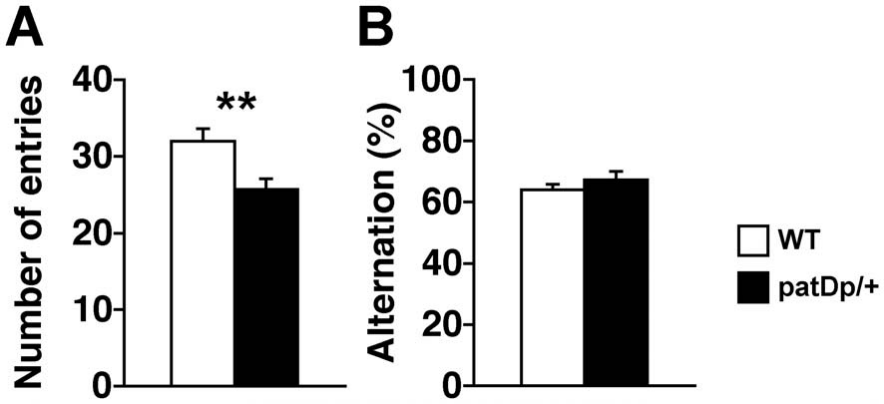
*patDp*/+ mice show decreased exploratory activity in Y-maze test. (**A**) The number of entries into each arm. (**B**) Percentage of traveled arm alterations. N = 22 for both genotypes. Error bars indicate SEM. **, *p*<0.01.

To examine exploratory behavior and working memory, we also performed the Y-maze test in these subjects. The number of entries into each arm was significantly decreased in *patDp*/+ mice compared with WT mice (Fig. 5A; *t*_42_ = 2.91; *p<*0.01). In contrast, no differences in alternation rates were observed (Fig. 5B; *t*_42_ = −0.95; *p = *0.35) over the 10-min test. These results suggest that although working memory in *patDp*/+ mice is not impaired they have decreased exploratory activity.

**Figure 6:**
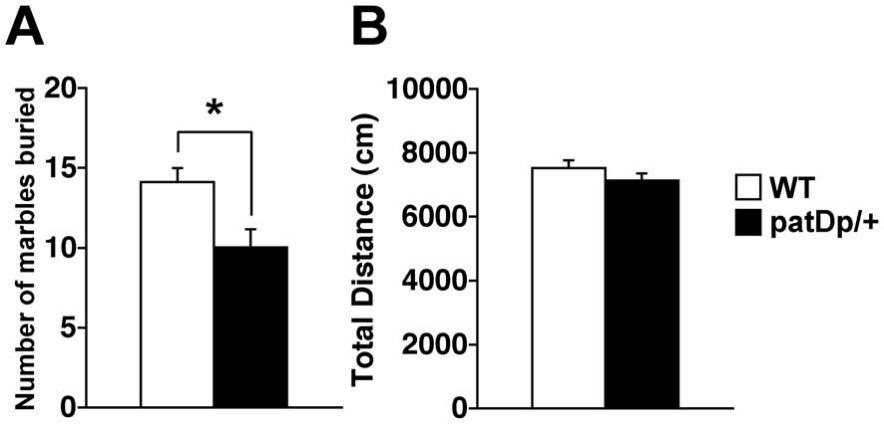
*patDp*/+ mice show less marble burying behavior. (**A**) The number of marbles buried for 30 min (*p*<0.01, *F_1,39_* = 7.63). The marble buried was defined as over 50% marble covered by bedding material. (**B**) Total distance traveled (*p* = 0.25, *F_1,39_* = 1.39). N = 19 for WT, 22 for *patDp/+* mice. Error bars indicate SEM. *, *p*<0.01.

Marble burying behavior is regarded as a rodent model for obsessive-compulsive disorder (OCD) or anxiety [16]. The number of marbles buried in *patDp/+* mice was significantly reduced compared with WT mice (Fig. 6A; *p<*0.01, *F*_1,39_ = 7.63), whereas no difference in locomotor activity between *patDp*/+ and WT mice was observed (Fig. 6B; *p = *0.25, *F*_1,39_ = 1.39). These results support that *patDp/+* mice have decreased exploratory activity.

### Decreased 5-HT and 5-HIAA in the *patDp*/+ adult brain

To establish the relationship between brain neurochemistry and behavioral disruptions in *patDp/+* mice, we quantified levels of biogenic monoamines *ex vivo*, including 5-HT and its major metabolite 5-hydroxyindoleacetic acid (5-HIAA), using HPLC in several adult brain regions. We specifically focused on 5-HT because it is one of the most notable molecules for the pathophysiology of ASDs [8], [9]. Levels of 5-HT and 5-HIAA were significantly decreased in several brain regions from *patDp*/+ mice (5-HIAA; OB, Ce, Mid, *t*_18_ = 2.72, 2.20, 2.99, *p*<0.05, 5-HT; OB, Mid, *t*_18_ = 2.23, 2.67, *p*<0.05). In contrast, there was no change in levels of the other monoamines, dopamine (DA) and norepinephrine (NE), compared with WT mice in any brain region. These findings suggest that the neurochemical differences between *patDp*/+ and WT mice are specific for the serotonergic system (Table 1). However, metabolic rates of 5-HT turnover, determined from the ratio of 5-HIAA to 5-HT, in *patDp*/+ mice were not altered compared with WT mice (Table 1), suggesting that monoamine oxidase (MAO) functions at a similar rate in both WT and *patDp/+* adult mice. Collectively, these results demonstrate alterations in the serotonergic system of *patDp/+* mice that may contribute to their behavioral phenotypes.

**Table 1:** Tissue levels of monoamines in adult WT and *patDp/+* mice.

Region and genotype		5-HT(pg/mg protein)	5-HIAA(pg/mg protein)	5-HT turnover(5-HIAA/5-HT)	DA(pg/mg protein)	DOPAC(pg/mg protein)	NE(pg/mg protein)
Cerebellum							
	WT	800.89±51.09	**610.68±32.47**	0.78±0.05	91.32±5.54	35.67±5.26	2988.23±71.28
	*patDp/+*	747.14±54.16	**533.36±13.65** *	0.74±0.05	85.99±2.69	31.20±1.47	2912.16±44.96
Midbrain							
	WT	**5723.94±181.88**	**4670.96±135.58**	0.82±0.03	2113.13±62.56	695.06±26.39	4731.57±105.54
	*patDp/+*	**5051.83±173.53** *	**4089.1±139.51** *	0.82±0.04	2985.01±931.77	808.40±193.80	4688.49±131.68
Olfactory bulb							
	WT	**4660.38±104.9**	**2015.87±145.69**	0.44±0.04	2630.63±113.94	770.08±53.47	3111.66±100.50
	*patDp/+*	**4266.45±142.64** *	**1590.04±57.23** *	0.38±0.02	2622.95±50.96	709.17±45.87	3244.77±63.60
Prefrontal cortex							
	WT	7093.56±142.73	1690.35(60.42	0.24(0.01	3208.95(1083.73	540.37(88.40	5771.15(123.21
	patDp/+	6846.74(102.99	1546.28(57.71	0.23(0.01	2151.43(595.04	373.67(60.14	5545.47(85.69
Pons and medulla							
	WT	4405.72(649.21	4354.89(294.4	1.28(0.24	1070.44(170.95	339.01(34.75	6086.68(204.58
	patDp/+	4359.11(620.47	4148.24(160.09	1.21(0.21	1041.80(149.70	337.42(20.21	6487.35(199.50

Values are means (SEM (n = 10, each genotype).

Difference between genotypes is noted

*p<0.05 by t-test.

### Disrupted developmental changes of 5-HT in *patDp*/+ mice

**Figure 7:**
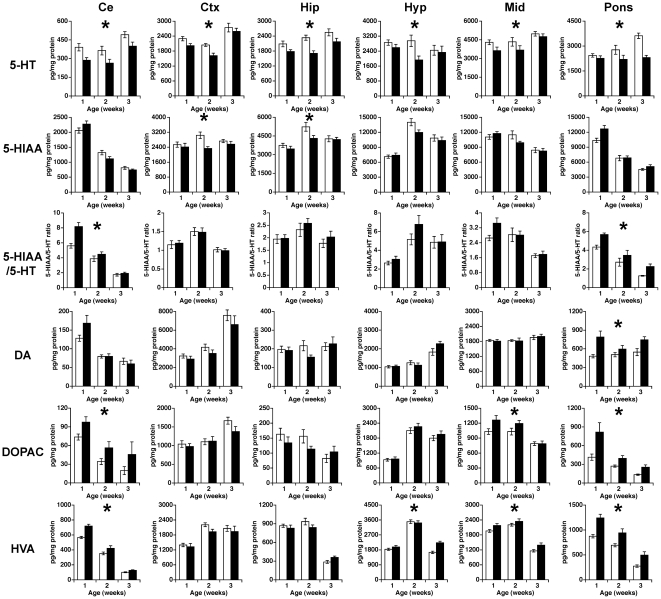
*patDp*/+ mice have decreased tissue 5-HT and increased tissue DA levels during postnatal development. Tissue levels of 5-HT, 5-HIAA, 5-HIAA/5-HT, DA, DOPAC and HVA in mice during the described developmental stages in the cerebellum (Ce), cerebral cortex (Ctx), hippocampus (Hip), hypothalamus (Hyp), midbrain (Mid), and pons and medulla (Pons). In WT mice, N  = 18–19 for postnatal (PND) 1 week old, 9–11 for PND 2 weeks old, and 7–14 for PND 3 weeks old. In *patDp/+* mice, N = 10–11 for PND 1 week old, 7–8 for PND 2 weeks old, and 4–8 for PND 3 weeks old. Error bars indicate SEM. *, *p*<0.05 (genotype effect).

ASDs are thought to be developmental disorders, and there are several neurodevelopmental disorders wherein subjects exhibit low brain levels of biogenic amines [17]. Thus, we performed a comprehensive investigation of brain monoamine levels in several brain regions of mice from postnatal weeks 1 to 3. Two-way ANOVA revealed that 5-HT concentrations in *patDp/+* mice tended to decrease in all brain regions tested over these developmental stages (genotype effect) (Fig. 7; 5-HT and Table S2). We also found that DA and its metabolites homovanillic acid (HVA) and 3,4-dihydroxyphenylacetic acid (DOPAC) tended to increase over these developmental stages in some brain regions of *patDp/+* mice, such as the pons and medulla (Fig. 7; DA, DOPAC, HVA and Table S2). Conversely, brain levels of NE and its metabolite 3-methoxy-4-hydroxyphenylglycol (MHPG) did not consistently increase or decrease over these developmental stages in *patDp/+* mice (Table S2). These results suggest that 5-HT signaling in the brains of *patDp*/+ mice is altered during these developmental stages.

## Discussion

Major autistic phenotypes such as impaired social interaction in the 3-chamber sociability test and perseverative responding in reversal learning tests have been reported in *patDp*/+ mice from either the C57BL/6J or 129S6 strains [13]. In the present study, we report additional behavioral abnormalities of C57BL/6J *patDp/+* mice. When conducting daily breeding procedures with these animals, we observed decreased movements and behavior. In support of this observation, *patDp/+* mice exhibited decreased exploratory activity in the open field test. Furthermore, this abnormal phenotype was observed in other behavioral tests, such as the Y-maze test. Collectively, these findings strongly suggest that *patDp/+* mice have decreased exploratory activity. In an evaluation of the underlying causes of decreased exploratory activity in *patDp/+* mice, motor tests showed that *patDp/+* mice possess normal motor coordination. This excludes the possibility that decreased exploratory activity in these animals resulted from dysfunctional motor coordination. Furthermore, lower exploratory activity in *patDp*/+ mice overcame increased appetite induced by 24 hours of food deprivation. As another example, in the marble burying test, *patDp/+* mice buried significantly fewer marbles than WT mice, but no difference in locomotor activity between *patDp*/+ and WT mice were observed. These results further suggest that decreased activity in *patDp*/+ mice may be elicited by anxiety induced by novel environmental conditions. However, decreased activity in *patDp*/+ mice was observed in their home cages and no significant differences were observed in the elevated plus maze test. Regarding these discrepancies, it is important to note that these measures are not inherently correlated and may reflect unique aspects of anxiety-like behaviors [18], [19]. However, reduced locomotor activity in the open field test was not reported in 129S6 mice [13]. This may be due to lower levels of spontaneous locomotor activity in 129S6 mice compared with C57BL/6J [20]. Interestingly, longer latencies to approach food during either eight-arm radial maze or T-maze tests have been observed in 129S6 mice [13]. Taken together, these findings indicate that the *patDp*/+ phenotype includes decreased exploratory activity, increased fear, or decreased motivation. In addition, patients with paternally derived duplication of 15q11-13 display motor coordination problems [7]. The mouse cerebellum is involved in exploratory behavior and motivational processes [21], and reduced exploration and stereotyped behavior in children with autism is linked with cerebellar hypoplasia [22]. Thus, the cerebellum of *patDp*/+ mice provides an intriguing target for further study.

In addition to abnormal monoamine concentrations in the cerebellum, we found decreased levels of both 5-HT and 5-HIAA in several brain regions of adult *patDp/+* mice and decreased levels of 5-HT during developmental stages from postnatal weeks 1 to 3 in all brain regions tested. The alterations in young mice were more prominent than the alterations in adult mice, suggesting that abnormalities of 5-HT signaling during childhood may contribute to the etiology of abnormal adult behaviors. Supporting this hypothesis, treating mice with SSRIs during postnatal days 4–21 influenced behavioral abnormalities (decreased exploratory activity in open field and elevated plus maze test, increased anxiety, depression related behaviors using novelty suppressed feeding, novelty induce hypophagia and shock escape test) when animals reached adulthood, and these abnormalities were not observed when adult animals were treated with SSRIs [23], [24]. Accordingly, 5-HT levels during childhood can influence adult behaviors and may contribute to abnormal behaviors in *patDp/+* mice.

In the context of ASDs, 5-HT is the most well established and extensively studied neurotransmitter [9]. For example, previous studies have shown that approximately one third of autistic children exhibited elevated blood levels of 5-HT, although this relationship is less definitive in adult autistics [8], [25]. Recently, a positive correlation of 5-HT levels of platelet-poor plasma and the brain has been shown in the rat autisitic model (rSey2/+) [26]. In addition, SSRIs can improve repetitive behavior, which is one of the core symptoms in ASDs [11]. Moreover, alterations in 5-HT synthesis capacity have been observed in the brains of patients with ASDs [27]. In this regard, recent studies on human genetics have revealed that the 5-HT transporter (5-HTT, also named SLC6A4) gene may contribute to increased risk for ASDs [28], [29]. The behavioral and neurochemical phenotypes identified in this study recapitulate those of animals deficient in 5-HTT or certain 5-HT receptors (e.g. 5-HT_1A_, 5-HT_2C_, and 5-HT_7_) [30]–[32]. Notably, 5-HTT−/− mutant mice or rats exhibit various behavioral defects that are similar to the phenotypes of *patDp/+* mice, such as decreased exploratory activity in the open field test, decreased marble burying behavior, lower basal activity in their home cages, and increased latency to feed in the novelty suppressed feeding test [33]–[38]. Interestingly, 5-HTT−/− mice also display less sociability and perseverance-like behaviors [37]–[39]. Moreover, 5-HTT mutations also result in approximately 70% reductions in levels of brain intracellular 5-HT [35], [40]. The similarity of these phenotypic and neurochemical findings to the results of the present study supports the hypothesis that disturbances in serotonergic signaling at early developmental stages might contribute to the behavioral phenotypes of *patDp/+* mice.

MBII-52 is a small nucleolar RNA that is located within the 15q11-13 region and is a regulator of 5-HT_2C_ receptor (5-HT_2C_R) mRNA post-transcriptional modifications. We have previously shown that MBII-52 is expressed at a two-fold higher level in the brains of *patDp/+* mice than WT mice. Furthermore, this increase in expression of MBII-52 had functional relevance because it increased intracellular calcium levels in neurons of *patDp/+* mice in response to a specific 5-HT_2C_R agonist, measured using an *in vitro* microspectrofluorimetric technique [13]. Whether MBII-52 regulates 5-HT_2C_R function *in vivo* remains to be determined as the editing ratio can be influenced by brain region and mouse strain specificities [41]. Although more work on the relationship between MBII-52 and RNA editing of the 5-HT_2C_R *in vivo* is required, several lines of evidence suggest that the 5-HT_2C_R can inhibit 5-HT neuronal activity and neurotransmission when expressed on GABAergic interneurons in the dorsal raphe nucleus, which are important modulators of 5-HT neurons [42]–[44]. Thus, *patDp/+* mice might have impaired local circuitry in the dorsal raphe nucleus, and this may result in the abnormal behaviors seen in *patDp*/+ mice.

Comprehensive quantification of monoamines also revealed increased DA signaling during developmental stages that may be relevant to the behavioral phenotypes of *patDp/+* mice. Many of the various functions and molecular pathways of DA in the mature brain have been reported [45]. However, recent studies reported biogenic amines including DA appear early during embryogenesis, before the onset of synaptogenesis, suggesting that they may play important roles in brain development [46]. *In vitro* studies demonstrated that during brain development DA acts as a promoter and an inhibitor of the number or length of branching neurites [47], [48]. Furthermore, DA signaling at earlier developmental stages may contribute to postnatal neurogenesis and migration of inhibitory interneurons [49]–[51]. As such, alterations in DA signaling during development might also result in the behavioral and neurochemical abnormalities in *patDp/+* mice.

In this study, we quantified tissue monoamine levels. Future studies should quantify extracellular monoamine levels using microdialysis procedures, and determine whether these abnormalities result from alterations in neurotransmitter release, reuptake, or synthesis. Furthermore, a precise analysis at a nuclear level, such as the raphe nucleus, would be informative. Such a precise study on 5-HT and DA levels at the neural circuit level would be necessary to decipher the neurochemical basis of abnormal behaviors in *patDp*/+ mice. Additionally, investigations of neurochemistry during embryonic development remain important for understanding when abnormalities of the 5-HT or DA pathways begin.

The results of the present study demonstrate behavioral and neurochemical abnormalities in *patDp/+* mice backcrossed to the C57BL/6J strain. These animals displayed lower locomotor/exploratory activity in most of the activity-related behavioral tests studied, and showed decreased 5-HT levels in several brain regions during development and at adulthood. We also found that tissue levels of DA and its metabolites were increased during development. Based on these findings, we propose that alterations in the serotonergic system at an early stage may contribute to abnormal behavioral phenotypes in adult *patDp/+* mice.

## Materials and Methods

### Animals and Experimental Design

We generated *patDp/+* mice as previously described [13]. They were bred for more than 10 generations on a C57BL/6J background. They were housed as a pair of 1 WT and 1 *patDp/+* mouse or 2 pairs of WT and *patDp/+* mice in a room with a 12-hour light/dark cycle (light on 7:00 a.m. and off 7:00 p.m.). All mice had access to food and water *ad libitum*. All experimental mice were male *patDp/+* ones, and age-matched (9–13 weeks old when behavioral experiments were started) WT littermates were used as controls. Twenty-two mice of each genotype were used for general health and neurological screens, neuromuscular examinations, light/dark transition test, open field test, elevated plus maze test, hotplate test, rotarod test, marble burying test, prepulse inhibition test, Y-maze test, tail suspension test, and contextual and cued fear conditioning test in the described order. All behavioral experiments were performed during the light phase (between 9:00 a.m. and 6:00 p.m.). Additionally, the numbers of 13–14 mice in each genotype were used for the novelty suppressed feeding test. For the home cage behavioral assay, 4 WT and 9 *patDp/+* mice were used. Finally, 10 adult mice of each genotype and 4–19 mice in each developmental stage of each genotype were used for HPLC analysis. The experimental procedures and housing conditions for animals were approved by the Animal Research Committee of Osaka Bioscience Institute (08-304) and the Committee of Animal Experimentation, Hiroshima University (A10-74). All efforts were made to minimize suffering during and after all surgeries.

### General health and neurological screen (GHNS)

A general health and neurological screen was conducted as previously described [52]. The righting, whiskers touch, and ear twitch reflexes were evaluated, and a number of physical features, including body weight, body temperature, and the presence of whiskers or bald hair patches, were recorded.

### Neuromuscular examination

Neuromuscular strength was examined by the grip strength and wire-hanging tests. The grip strength meter (O'Hara & Co., Tokyo, Japan) was used to assess forelimb grip strength. Mice were lifted and held by their tail so that they could grasp a wire grid with their forepaws. Mice were then gently pulled backward by the tail with their posture parallel to the surface of the table until they released the grid. The peak forelimb grip force applied by the mice was recorded in Newtons [19]. Each mouse was tested three times and the greatest value measured was used for statistical analysis. In the wire hang test, mice were placed on a wire mesh that was then inverted and waved gently, so that the subject gripped the wire. Latency to fall within 60 sec was recorded by counting manually.

### Light/dark transition test

The apparatus used for light/dark transition test consisted of a cage (21×42×25 cm) divided into two sections of equal size by a partition with a door (O'Hara & Co.) [53]. One section was brightly illuminated (390 lux), whereas the other section was dark (2 lux). Mice were placed into the dark side of the apparatus, and allowed to move freely between the two sections for 10 min, while the door remained open. The total number of transitions, time spent in each section, initial latency to the light section, and distance traveled were recorded automatically using Image LD software. On-line material describing this method is available [53].

### Open field test

Locomotor activity was measured using an open field test. Each subject was allowed to move freely in the open field apparatus (40×40×30 cm; Accuscan Instruments, Columbus, OH, USA) equipped with photocells (beam spacing 2.5 cm, beam diameter 4 mm, beam frequency 50 cycles/s). Total distance traveled, vertical activity (rearing measured by counting the number of photobeam interruptions), time spent in the center area of the open field, and stereotypic counts were recorded using the VersaMax system (Accuscan Instruments). The center area was defined as an inner section 1 cm away from each of the walls. If the beam at the edge of the open field, 1 cm apart from the wall, was not interrupted, mice were considered to be in the center area. If a mouse broke the same beam (or set of beams) repeatedly, it was considered to be exhibiting stereotypic activity. This activity is often observed during grooming or head bobbing behaviors. Stereotypic counts are the number of beam breaks that occur during any period of stereotypic activity. Data were collected for 120 min.

### Elevated plus maze test

The elevated plus maze consisted of two open arms (25×5 cm) and two enclosed arms of the same size, with 15 cm high transparent walls. The arms and central square were made of white plastic plates, and were elevated to a height of 55 cm above the floor. To minimize the likelihood of animals falling from the apparatus, 3-mm high Plexiglas ledges surrounded the open arms. Arms of the same type were arranged at opposite sides to each other. Each mouse was placed in the central square of the maze (5×5 cm) facing one of the enclosed arms. Time spent in each arm was recorded during the 10-min test period. Data acquisition and analysis were performed automatically using Image EP software.

### Hot plate test

The hot plate test was used to evaluate nociception. Mice were placed on a 55.0±0.3°C hot plate (Columbus Instruments, Columbus, OH, USA) and latency to the first hindpaw response was recorded by counting manually. The hindpaw response was either a foot shake or a paw lick. Each mouse is tested once.

### Rotarod test

Motor coordination and balance were tested using the rotarod test. This test was performed by placing a mouse on a rotating drum (3 cm diameter; Accelerating Rotarod, UGO Basile, Varese, Italy) and measuring the latency (seconds) of each subject to fall from the rod. Six trials were performed and the speed of the rotarod was increased from 4 to 40 rpm over a 5-min period.

### Marble burying behavior test

Mice were individually placed in transparent polycarbonate cages (12×27×9 cm) with a 5-cm layer of fine bedding material and 25 equally spaced glass marbles (1.5 cm in diameter). The number of buried marbles and the total distance traveled were recorded with a video camera for 30 min. When half of the marble was in the fine bedding material, it was operationally defined as buried. Three WT mice jumped out of the test cage during this test and were omitted from subsequent data analysis.

### Startle response/prepulse inhibition tests

Startle responses and prepulse inhibition of the startle responses were measured using an automatic startle reflex measurement system (O'Hara & Co.). A test session began by placing a mouse in a Plexiglas cylinder, and left undisturbed for 10 min. The startle stimulus was a broadband white noise that lasted 40 msec for all trial types and the startle response was recorded during this time period (measuring the response every 1 msec) without the prepulse stimulus. The prepulse inhibition response was recorded for 140 msec starting with the onset of the prepulse stimulus. The background noise level in each chamber was 70 dB. The peak startle amplitude recorded during the 140 msec sampling window was used as the dependent variable. A test session consisted of 6 trial types (i.e. two types for startle stimulus only trials, and four types for prepulse inhibition trials). The intensity of the startle stimulus was 110 or 120 dB. The prepulse was presented 100 msec before the startle stimulus with an intensity of 74 or 78 dB. Four combinations of prepulse and startle stimuli were employed (74–110, 78–110, 74–120, and 78–120). Six blocks of the 6 trial types were presented in pseudorandom order, such that each trial type was presented once within a block. The average intertrial interval was 15 sec (range: 10–20 sec).

### Y-maze test

Exploratory activity was measured using a Y-maze apparatus (arm length: 40 cm, arm bottom width: 3 cm, arm upper width: 10 cm, height of wall: 12 cm). Each subject was placed in the center of the Y-maze field. The number of entries and alterations were recorded using a modified version of the Image EP program. Data were collected for 10 min.

### Tail suspension test

The tail suspension test was performed following previously described procedures [54]. Mice were suspended 30 cm above the floor in a visually isolated area by adhesive tape placed approximately 1 cm from the tip of the tail. Their behavior was recorded over a 10 min test period. Data acquisition and analysis were performed automatically using Image TS software. A single WT and a single *patDp/+* mouse fell from the adhesive tape and their data were omitted from subsequent analysis.

### Contextual and cued fear conditioning

Each mouse was placed in a test chamber (26×34×29 cm) within a larger sound-attenuated chamber (O'Hara & Co.) and allowed to explore freely for 2 min. A 60 dB white noise, which served as the conditioned stimulus (CS), was presented for 30 sec. Next, a mild (2 sec, 0.5 mA) foot shock, which served as the unconditioned stimulus (US), was presented immediately after the CS. Two more CS-US pairings were presented with a 2 min interstimulus interval. Context testing was conducted 24 h after conditioning in the same chamber. Cued testing with altered context was conducted after conditioning using a triangular box (35×35×40 cm) made of white opaque Plexiglas, which was located in a different room [55]. Data acquisition, control of stimuli (i.e. tones and shocks), and data analysis were performed automatically using Image FZ software. Images were captured at 1 frame per sec. For each pair of successive frames, the amount of area (in pixels) the mouse moved was measured. When this area was below 20 pixels, the behavior was operationally defined as ‘freezing’. When the amount of area equaled or exceeded the threshold, the behavior was defined as ‘non-freezing’. The optimal threshold (amount of pixels) for determining freezing versus non-freezing was calibrated to human observation of the behavior. Freezing that lasted less than 2 sec was not included in the analysis.

### Novelty suppressed feeding test

This test was performed according to the previous study [56] with some modifications. Twenty-four hours before the test, each mouse was weighed and then deprived of all food in their home cage. Prior to test, a food pellet was placed on a round filter paper (10 cm) and positioned in the center of the apparatus (40×40×40 cm). The subject mice were weighed again and the difference of body weight loss was assessed. The test began immediately after each subject was placed in the corner of the apparatus and latency to feed the pellet was measured by counting manually. The cutoff time of this test was set to 5 min. Immediately, after attempting to feed or 5 min, each subject was transferred to its home cage and the amount of food eaten was measured by weighing pre- and post fed food pellet for 5 min to evaluate their appetite.

### Circadian rhythms of locomotor activity

Circadian rhythms of locomotor activity were analyzed as previously described [57]. Each mouse was individually housed for 2 weeks in a 12 hour light-dark cycle condition (LD), and then for 2 weeks in constant darkness (DD). Locomotor activities were monitored with an infrared locomotor recording apparatus (Biotex, Kyoto, Japan) by recording activity in 1-min bins. The data from the final week of LD were used to calculate spontaneous activities of *patDp/+* and WT mice. Circadian period was estimated in each mouse from the last 5 days of locomotor activity under DD.

### Monoamine quantification in brain tissues

Tissue concentrations of biogenic monoamines were analyzed after dissection in various brain regions, depending on the age of the mouse. In adults, monoamines were measured in the cerebellum (Ce), midbrain (Mid), olfactory bulb (OB), prefrontal cortex (PFC), and pons and medulla (Pons). In young mice, monoamines were analyzed in the Ce, Mid, Pons, cerebral cortex (Ctx), hippocampus (Hip), and hypothalamus (Hyp). Brain tissue was homogenized in 0.2 M ice-cold perchloric acid and the homogenates were cooled on ice for 30 min to deproteinize. The homogenates were centrifuged at 20,000× g for 15 min at 0°C. Then, the pH of the supernatant was adjusted to approximately 3.0 by adding 1 M sodium acetate, and the precipitate was used for protein quantification. The samples were filtered through a 0.45 µm filter (Millipore, Billerica, MA, USA) and centrifuged at 500× g for 15 min at 4°C. Next, 30–40 µl of filtrate was loaded into a high performance liquid chromatography (HPLC) system (Eicom, Kyoto, Japan). The HPLC system had a 150×3 mm octadecyl silane column (SC-5ODS, Eicom), and an electrochemical detector (HTEC-500, Eicom) set to an applied potential of +750 mV versus an Ag/AgCl reference analytical electrode. The change in electric current (nA) was recorded using a computer interface. The mobile phase was composed of aceto-citric acid buffer (pH 3.5, 0.1 M), methanol, sodium-1-octane sulfonate (0.46 M), and disodium ethylenediaminetetraacetic acid (0.015 mM) [830: 170: 1.9: 1]. The flow rate was 0.5 ml/min. Protein quantification was performed using a BCA method according to manufacturer's protocol (Sigma, St. Louis, MO, USA).

### Data analysis

Behavioral data were obtained automatically by customized (O'Hara & Co.) applications that were based on a public domain NIH Image program and the ImageJ program. Statistical analysis was conducted using StatView Ver 5.0 (SAS Institute, Cary, NC, USA). Data were analyzed by two-way analysis of variance (ANOVA), two-way repeated-measures ANOVA, or unpaired *t*-test. Unless otherwise noted, the *F* and *p* values are for the genotype effect. The criterion for significance was set at *p<*0.05. Kaplan-Meier survival analysis was used to analyze data from the novelty suppressed feeding test because these data did not have a normal distribution. Mantel-Cox log rank test was used to evaluate differences between genotypes in this test.

## Supporting Information

Table S1 **Results of a comprehensive behavioral battery in WT and** ***patDp/+*** **mice.**
(XLS)

Table S2 **Developmental changes in tissue levels of monoamines in WT and** ***patDp/+*** **mice.**
(XLS)
